# An overview of compensated work-related injuries among Korean firefighters from 2010 to 2015

**DOI:** 10.1186/s40557-018-0268-2

**Published:** 2018-09-03

**Authors:** Hyung Doo Kim, Yon Soo An, Dong Hyun Kim, Kyung Sook Jeong, Yeon Soon Ahn

**Affiliations:** 1 Department of Preventive Medicine and Institute of Occupational and Environmental Medicine, Wonju College of Medicine, Yonsei University, 20 Ilsan-ro, Wonju, Gangwon-do 26426 Republic of Korea; 2 Department of Occupational and Environmental Medicine, Inha University Hospital, 27 Inhang-ro, Jung-gu, Incheon, 22332 Republic of Korea; 3 Department of Occupational and Environmental Medicine, Soonchunhyang University Cheonan Hospital, 31 Suncheonhyang 6-gil, Dongnam-gu, Cheonan-si, Chungcheongnam-do 31151 Republic of Korea; 4 Department of Occupational and Environmental Medicine, Dongguk University Ilsan Hospital, 27 Dongguk-ro, Ilsandong-gu, Goyang, 10326 Republic of Korea; 5 Department of Occupational and Environmental Medicine, Hallym University Sacred Heart Hospital, 22 Gwanpyeong-ro 170beon-gil, Dongan-gu, Anyang-si, Gyeonggi-do 14068 Republic of Korea

**Keywords:** Firefighter, Korean, Occupational injury, Occupational accident, Occupational environment, Work-related injury, Industrial accident

## Abstract

**Background:**

Although one in two firefighters in South Korea have experienced work-related injuries, there are few studies which show the overview description on work-related injuries and its analysis regarding such causes. Therefore, we aimed to show the overview of compensated work-related injuries in order to serve fundamental data for establishing prevention policies on work-related injuries for Korean firefighters.

**Methods:**

We requested the all claimed work-related injury data of Korean firefighters from 2010 to 2015 to the Korean National Fire Agency (NFA). The data from NFA including 2457 claimed cases was analyzed and we confirmed, 2154 approved work-related injuries for the kinds of job activities, cause of accident and type of injuries. Among 2154 approved cases, we analyzed more variables for the sex, age, and job duration of 1344 compensated cases through served text file on summary of accident.

**Results:**

The Government Employees Pension Service (GEPS) recognized 2154 (87.7%) approved work-related injuries among 2457 claimed cases. The incidence of work-related injuries per 1000 firefighters was 9.8 persons. By region, the incidence of work-related injuries per 1000 firefighters ranged from a maximum of 14.5 to a minimum of 4.0. The most common job activity caused the accident was fire suppression (18.0%), followed by Emergency medical services (EMS) (17.5%) and training (10.7%). The most common cause of these accident was movement imbalance (30.3%), followed by falls (18.9%) and traffic accident (13.4%). In these work-related injuries, sprains and bruises were the most common type of injury (27.2%), and the most commonly injured body site was the upper and lower back (25.3%). Data from identified 1344 firefighters showed that 1264 (94.0%) were male and 80 (6.0%) were female. Age group was the highest in the 40s with 623 cases (46.4%), and job duration was the highest with 650 cases in 5–10 years (48.4%).

**Conclusion:**

In this study, we could obtain the preliminary data necessary to establish preventive measures, including the cause of accident and region with high accident rates. However, the number of applications for compensated injuries was very small compared to the frequency of injuries found in previous studies. The lack of appropriate treatment suggested that many firefighter injuries can become chronic. In this study, we suggest that it is necessary to introduce an injury monitoring system and improve the accessibility of compensated injuries.

**Trial registration:**

CR318031. Registered 20 June 2018.

## Background

The incidence of work-related injuries among firefighters in South Korea is devastating, as highlighted in a study of Korean firefighters that surveyed via mail. According to the survey, 1216 (12.0%) of 10,127 fire suppression workers, 586 (18.5%) of 3169 EMS workers, and 299 (17.8%) of 1681 officers, had experienced one or more workplace injuries during the previous 12 months [1]. The risk of non-fatal injuries in firefighters was 1.4–7.4 times higher than that of other occupations in United States [2]. Although the occupational risk to firefighters is higher than normal population, the rate of on-duty deaths per 100,000 firefighters in South Korea is 2 times higher than that of United States (33 Korean firefighters died on duty from 2010 to 2014), suggesting that they are exposed to considerable occupational risk [3].

According to the Industrial Accident Analysis Data from 2010 to 2014 released by the Korea Occupational Safety & Health Agency, the average incidence of all industrial accidents per 1000 persons in South Korea is 6.0. The incidence of work-related injuries per 1000 firefighters is 1.6 times the average incidence of all industrial accidents per 1000 persons in South Korea. In addition, firefighters have a higher incidence of musculoskeletal diseases and noise-induced hearing loss due to the nature of the work, which exposed to various harmful factors than other occupations [4–8]. Although there is growing interest in work-related injuries among firefighters, there are only few studies that provide an overview of injuries to firefighters. We aimed to grasp the overview of compensated work-related injury among firefighters using the claimed data collected by the Korea Government Employee Pension Service (GEPS) from 2010 to July 2015, and use as preliminary data for establishing work-related injury prevention policies among firefighters.

## Methods

### Study participants

We requested the all claimed work-related injury data of Korean firefighters from 2010 to 2015 to the NFA. We analyzed 2154 approved firefighter cases from among 2457 claimed firefighter cases who submitted compensation applications for work-related injuries to the GEPS from January 2010 to July 2015. Only injury cases were included in this analysis.

### Variables

We analyzed job activity, accident cause, injury type, the region of fire station and recuperation duration among 2154 approved cases. Among 2154 approved cases, we could get more information about age, sex and job duration in 1344 compensated cases through the text file for summary of accident. In the statistical data of the NFA, the incidence of work-related injuries were analyzed by referring to the number of firefighters and the frequency of dispatch [9–13].

Compensated cases were classified based on four categories: sex (male, female), age (20–29, 30–39, 40–49, 50–59 years, 60 years and over), region of fire station (Seoul, Busan, Daegu, Incheon, Gwangju, Daejeon, Ulsan, Gyeonggi, Gangwon, Chungbuk, Chungnam, Jeonbuk, Jeonnam, Gyeongbuk, Gyeongnam, and Jeju), and job duration (0–5, 5–10, 10–20, 20–30, 30 years and over).

The causes of accident were classified as falling from a height, slips, falling beneath, coming into contact with an object, being struck with an object, being trapped, involvement in a structural collapse, movement imbalance, hazardous and dangerous material exposure, fire, violence, traffic accident, and others based on the Industrial Accident Cause Statistics of the Ministry of Employment and Labor [13]. The incidence of work-related injuries is expressed in a number of injuries per 1000 firefighters. The job activities were classified as fire suppression, emergency medical services (EMS), rescue, training, respond/return, commuting, office work or facility repair, outside work service, athletic events, and others. The type of injuries was classified as sprains and bruises, fractures, ligament injury and dislocations, nerve injury, muscle injury, laceration or injury from a bite, burns, amputations, poisoning, hearing loss, visual impairment, frostbite, and others. Body parts of injuries were classified as head, neck, shoulder, arm, forearm and hand, trunk, upper and lower back, upper leg and knee, lower leg and knee, and others.

### Statistical analysis

The descriptive analyses were performed for sex, age, job duration, region of fire station, job activity, accident cause, and injury type using the SPSS version 21.0 software program.

## Results

### Incidence of work-related injuries

**Fig. 1:**
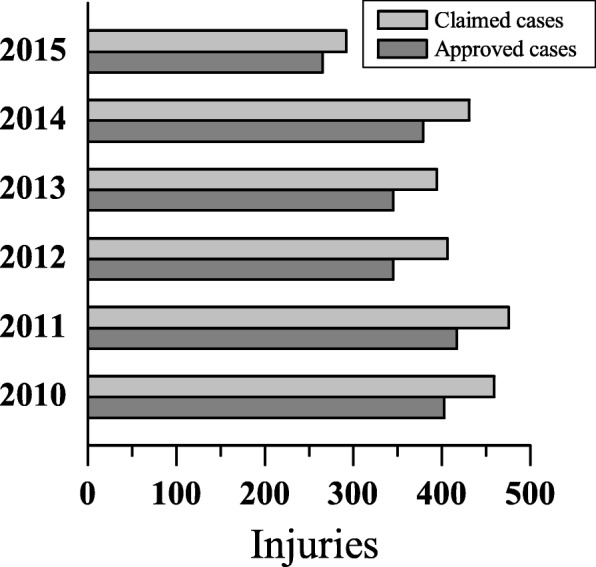
Number of claimed or approved work-related injuries by year. Gray bars mean total number of claimed cases, while dark gray is the total number of approved cases

The GEPS recognized 2154 (87.7%) approved work-related injuries among 2457 claimed cases. The proportion of compensated work-related injuries per year was as follow: 87.8% in 2010, 87.8% in 2011, 85.0% in 2012, 87.6% in 2013, 87.9% in 2014 and 90.8% from January to July 2015 (Fig. 1).

The incidence of work-related injuries per 1000 firefighters was 9.8, from 8.7 in 2013 to 11.0 in 2011. Based on the analysis of the region of fire station, the Gyeonggi Province was the largest proportion of compensated cases (415, 19.3%), followed by 382 (17.7%) in Seoul, and 161 (7.5%) in Daegu. The incidence of work-related injuries per 1000 firefighters was the highest in Daegu area (14.4), followed by Jeonbuk (13.7), and Gangwon Province (12.5). The lowest in Gwangju (4.0), followed by Gyeongbuk (4.4), and Jeonnam (7.1) (Table 1).

**Table 1:** The incidence of approved work-related injuries per 1000 firefighters by the region and year (2010 to 2014)

Variables		*2010*			*2011*			*2012*			*2013*			*2014*			*Total*		
		*N* ^a^	*n* ^b^	*‰* ^c^	*N* ^*a*^	*n* ^b^	*‰* ^c^	*N* ^a^	*n* ^b^	*‰* ^c^	*N* ^a^	*n* ^b^	*‰* ^c^	*N* ^a^	*n* ^b^	*‰* ^c^	*N* ^a^	*n* ^b^	*‰* ^c^
Region	Seoul	5800	67	11.6	6051	90	14.9	6359	55	8.7	6501	51	7.8	6461	70	10.8	30,839	333	10.7
	Busan	2546	34	13.4	2546	28	11.0	2559	26	10.2	2594	20	7.7	2635	20	7.6	12,752	128	9.9
	Daegu	1878	34	18.1	1959	24	12.3	1959	18	9.2	1985	31	15.6	1975	34	17.2	9615	141	14.5
	Incheon	2222	16	7.2	2237	18	8.1	2246	7	3.1	2255	27	12.0	2236	27	12.1	11,101	95	8.5
	Gwangju	1092	6	5.5	1092	6	5.5	1092	5	4.6	1115	3	2.7	1108	2	1.8	5477	22	4.0
	Daejeon	1129	11	9.7	1136	17	15.0	1139	13	11.4	1158	12	10.4	1157	10	8.6	5656	63	11.0
	Ulsan	717	12	16.7	777	11	14.2	835	8	9.6	835	3	3.6	828	10	12.1	3948	44	11.0
	Gyeonggi	5538	81	14.6	5957	77	13.0	6176	62	10.0	6176	70	11.3	6418	67	10.4	29,908	357	11.8
	Gangwon	2277	28	12.9	2203	30	13.6	2253	22	9.8	2290	29	12.7	2291	31	13.5	11,074	140	12.5
	Chungbuk	1378	16	11.6	1382	16	11.6	1456	13	8.9	1479	13	8.8	1462	6	4.1	7093	64	8.9
	Chungnam	2033	21	10.3	2059	17	8.3	1986	20	10.1	2270	18	7.9	2270	10	4.4	10,532	86	8.1
	Jeonbuk	1970	21	10.7	1970	23	11.7	1970	32	16.2	1970	27	13.7	1927	31	16.1	9673	134	13.7
	Jeonnam	2058	14	6.8	2062	17	8.2	2066	12	5.8	2066	10	4.8	2022	20	9.9	10,201	73	7.1
	Gyeongbuk	2570	13	5.1	2747	9	3.3	2934	13	4.4	3033	13	4.3	2976	14	4.7	14,198	62	4.3
	Gyeongnam	2764	22	8.0	2764	27	9.8	2780	28	10.1	2823	10	3.5	2796	20	7.2	13,820	107	7.7
	Jeju	594	7	11.8	627	7	11.2	647	11	17.0	647	7	10.8	647	7	10.8	3123	39	12.3
Total		36,711	403	11.0	37,826	417	11.0	38,557	345	8.9	39,519	344	8.7	40,406	379	9.4	191,131	1888	9.8

Firefighters working in Sejong are included in Chungnam. ^a^Total number of firefighters; ^b^Number of approved work-related injuries; ^c^Proportion of injuries (per 1000 persons)

From 2010 to 2014, the monthly incidence of work-related injuries was high during the period from April to June; 186 in April (9.8%), 197 in May (10.4%), and 173 in June (9.2%). The number of work-related injuries per 1000 dispatches was 8.9. February was the lowest at 6.0, and August was the highest at 11.5. No increase in incidence with increasing dispatch was observed (Table 2).

**Table 2:** The incidence of approved work-related injuries per 1000 dispatches by the month

Variable	Jan.	Feb.	Mar.	Apr.	May.	Jun.	Jul.	Aug.	Sep.	Oct.	Nov.	Dec.	Total
Compensated cases	158	122	163	186	197	173	158	161	136	158	146	131	1889
Total Dispatches	21,774	20,299	22,186	20,085	18,166	16,053	14,024	14,007	13,605	15,821	16,483	19,550	212,053
work-related injuries per 1000 Dispatches	7.3	6.0	7.3	9.3	10.8	10.8	11.3	11.5	10.0	10.0	8.9	6.7	8.9

### Burden of work-related compensated injuries (recuperation duration and compensation cost)

**Fig. 2:**
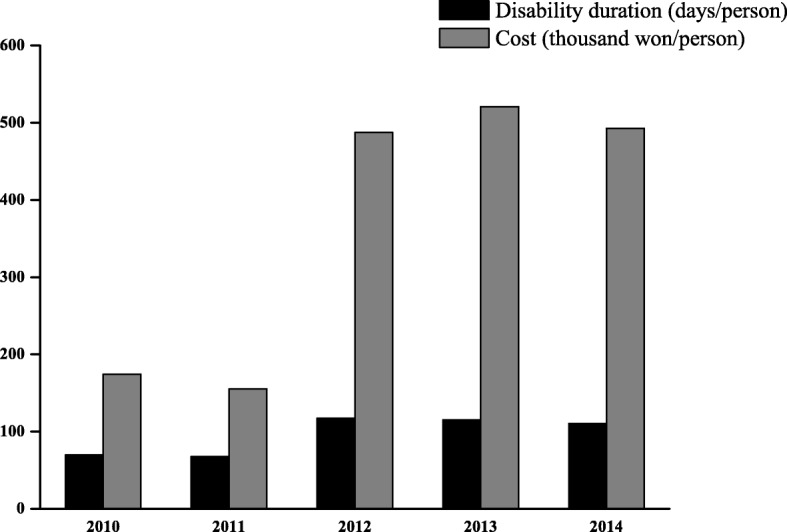
The average recuperation duration (days/person) and compensation cost (thousand won/person) per firefighter

The average recuperation duration during the study period was 96.4 days. The recuperation duration was the longest at 115.2 days in 2013 (Standard deviation, SD: 104.0), and the shortest was 67.7 days in 2011 (SD: 54.8). The per-claim average compensation cost of injury to firefighters was 358,780 won. In 2013, it was the highest at 520,926 won (SD: 1,028,345), the lowest at 155,049 won in 2011 (SD: 648,347) (Fig. 2).

### Characteristics of compensated work-related injuries

We analyzed 2154 approved work-related injuries for job activities, causes of accident and type of injuries (Table 3).

**Table 3:** Recuperation duration and amount of payment according to type of injuries and injured body sites

Variable			Recuperation duration (days)			Compensation (thousand won)		
	*N*	%	Mean	Median	Max	Mean	Median	Max
All	2154	100	96.4	60	1183	358.8	19.3	14,889.8
Sprains and bruises	586	27.2	52.9	60	228	88.0	0	2386.9
Fractures	501	23.3	110.0	90	730	397.6	44.9	11,416.0
Ligament injury and dislocations	421	19.5	101.3	69	1183	527.8	52.9	6429.9
Nerve injury	177	8.2	90.9	60	816	544.5	90.4	6407.1
Laceration or injury from bite	115	5.3	68.6	60	236	106.9	9.7	1733.9
Muscle injury	99	4.6	82.7	60	366	294.3	16.6	3096.0
Burns	94	4.4	89.3	60	383	626.0	67.5	14,889.8
Amputations	12	0.6	86.2	61	180	186.0	0	2094.2
Poisoning	8	0.4	64.9	60	99	110.0	23.5	450.7
Hearing loss	8	0.4	112.6	60	464	121.7	0	551.9
Visual impairment	4	0.2	46.8	60	60	4.7	0	18.7
Frostbite	1	0.0	60.0	60	60	0	0	0
Others	128	5.9	278.8	92	883	773.8	14.9	11,343.6
Upper and lower back	544	25.3	73.7	60	816	296.2	17.9	7965.7
Upper leg and knee	368	17.1	94.0	63	1183	477.0	30.1	6429.9
Lower leg and foot	267	12.4	98.6	78	726	416.7	64.2	11,416.0
Head	262	12.2	103.0	60	883	265.5	0	14,889.8
Forearm and hand	219	10.2	89.7	60	366	223.6	30.8	4139.6
Shoulder	128	5.9	82.9	60	309	436.2	10.1	3702.2
Trunk	107	5.0	100.1	60	383	389.0	0	4980.0
Neck	79	3.7	65.9	60	263	79.5	0	1283.2
Arm	68	3.2	99.1	72	320	367.5	36.3	8105.7
Others	112	5.2	239.3	90	730	693.0	13.8	11,343.6

**Fig. 3:**
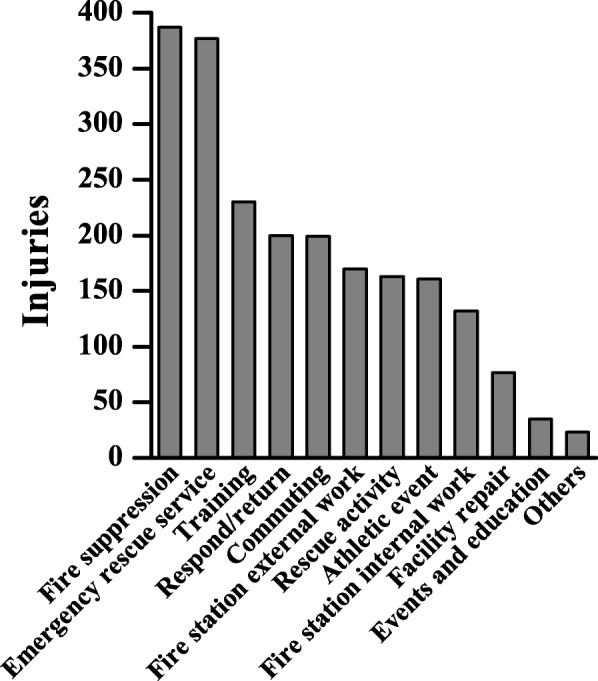
Number of approved work-related injuries by duty. The number of work-related injuries among firefighters was highest in fire suppression, followed by emergency rescue activities, and training. All natures of duty conducted only injured situation

**Fig. 4:**
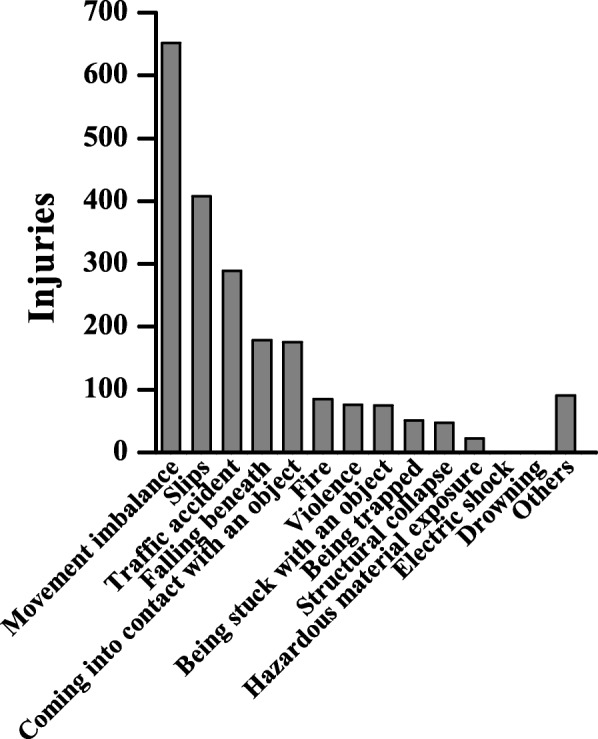
Cause of injuries in firefighters. The most common cause of injury was movement imbalance, followed by slips and traffic accidents. All natures of multiple injury were counted only most severe site

The most common job activity at the time of accident was fire suppression (*n* = 387, 18.0%), followed by rescue activities (*n* = 377, 17.5%), training (*n* = 230, 10.7%), respond/return (*n* = 200, 9.3%), and commuting (*n* = 199, 9.2%) (Fig. 3). In terms of accident cause, movement imbalance was the most common cause (*n* = 652, 30.3%), followed by falls (*n* = 408, 18.9%), traffic accidents (*n* = 289, 13.4%), falling from a height (*n* = 179, 8.3%), and coming into contact with an object (*n* = 176, 8.2%) (Fig. 4).

In terms of injury type, sprains and bruises were the most common (*n* = 586, 27.2%), followed by fractures (*n* = 501, 23.3%), ligament injuries and dislocations (*n* = 421, 19.5%), nerve injuries (*n* = 177, 8.2%), and sharps or bite injuries (*n* = 115, 5.3%).

The upper and lower back were the most commonly injured sites (*n* = 544, 25.3%), followed by the upper leg and knee (*n* = 368, 17.1%), the lower leg and foot (*n* = 267, 12.4%), the head (*n* = 262, 12.2%), and the forearm and hand (*n* = 219, 10.2%).

### The characteristics of identified firefighters

Among 2154 approved work-related injuries, only 1344 firefighters were identified such variables as sex, age and job duration through the text file for accident summary (Table 4).

**Table 4:** The characteristics of a part of study subjects

Variable	*n*	%	
Sex			
Male	1264	94.0	
Female	80	6.0	
Age (years)			
20–29	41	3.1	
30–39	477	35.5	
40–49	623	46.4	
50–59	200	14.9	
≥ 60	3	0.2	
Job duration (years)			
< 5	109	8.1	
5–10	290	21.6	
10–20	650	48.4	
20–30	258	19.2	
≥ 30	37	2.8	

1264 (94.0%) were male and 80 (6.0%) were female. The average age of firefighters was 47.7. The most common age group was 40–49 (46.4%), followed by 30–39 (35.5%). Job duration was average 14.2 years. 10–20 years were the largest with 650 (48.4%), followed by 5–10 years with 290 (21.6%). A further analysis of the 10–20 years’ job duration firefighter group, which accounted for a large proportion, did not show any difference from the other groups.

## Discussion

The significance of this study is the analysis of the incidence, regional differences, cause of accident, and job activity through compensated work-related injury data of NFA for about 5 years in all firefighters in Korea.

The incidence of compensated work-related injuries per 1000 firefighters was just 9.8 persons. However, there is a large difference between this finding and the results of a previous survey of work-related injuries of Korean firefighters that reported 116 cases per 1000 firefighters within the preceding 12 months [1]. This difference is likely due to a number of firefighters not claiming compensation for work-related injury compensation even when they had experienced a work-related injury. 33 Korean firefighters died on duty from 2010 to 2014. The rate of on-duty deaths per 100,000 firefighters in Korea is 83.2, compared to 35.9 in US firefighters [3]. This suggests that firefighters in Korea are exposed to more dangerous work environments. However, the accident rate is the opposite, the accident rate in the US in 2013 was 15.1%, while the rate of injury in Korea was 0.7% in the same year. That means that a large number of firefighters compensated injury does not apply for non-fatal work-related injury [3]. The most common reason for not reporting work-related injuries was because of mild disease [14]. Furthermore, due to concerns that reporting work-related injuries would be a burden to their colleagues or to the fire service, a failure to recognize a work-related injury or ignorance of the application procedure may have resulted in late reporting of work-related injuries, and neglect and aggravation of the injury [15]. Consequently, the work-related injury may have a long duration, and injury sequelae may require treatment for several days to several years, even after being treated as a compensated work-related injury. Therefore, systematic injury report systems and treatment systems for managing work-related injuries are needed. This provides additional insight into mild or severe but not reported injuries. There is a difference in incidence by region. To find out the cause, it is necessary to study the difference in the number of firefighting activities, preventive measures, and accessibility of compensated injuries. Preventive measures can be established through these studies.

Analysis of recuperation duration and compensation cost shows that these are constantly increasing. Although the increase in medical expenses every year can be judged by an increase in medical prices, a sharp increase from 2010 to 2015 can be interpreted as occupational injuries becoming more severe and becoming chronic. It is reported that during the recuperation duration, many firefighters fail to fully recover, paying their personal costs and receiving additional treatment [16]. Therefore, it is necessary to prevent deterioration of injury through early detection and early treatment, and to minimize the loss of firepower through quick recovery for them to return to work.

The most common job activity that caused the accident was fire suppression (*n* = 387, 18.0%), followed by EMS (*n* = 377, 17.5%), and training (*n* = 230, 10.7%). There was a high probability that firefighters may be injured during fire dispatch since, according to the statistics data released by the NFA, fire dispatches, rescue activity, and EMS accounted for 1.6%, 18.6% and 79.8% of all the dispatches from 2010 to 2014, respectively [9–13]. 927 compensated cases (43.0%) occurred in direct firefighting activities such as fire suppression, EMS, rescue, and 391 compensated cases (18.2%) occurred in training and athletic events. This indicates that current training has become an important cause of work-related injuries among firefighters, and in conflict with its original purpose of preventing injuries and enhancing job performance. And effective training methods to prevent injuries occurring during training or athletic events [17–19].

Also, 199 cases (9.2%) of injuries were caused by traffic accidents. Further studies are needed to determine whether the occurrence of a traffic accident is a work-related fatigue due to shift work. Previous studies have also shown that decreased concentration was associated with increased occupational accidents or traffic accidents [20–23]. Therefore, it is necessary to study the fatigue caused by the shift work of the firefighters and their effects.

We reviewed the compensated work-related injuries applications received by the NFA and analyzed the causes of the injury in detail. The most common injuries pertained to musculoskeletal injuries of the lower back or lower legs, caused by a movement or a fall. Therefore, preventive measures against frequent injuries should be given priority. Programs should be developed to improve the strength and flexibility or frequent injured sites in firefighter training [17–19]. In addition, financial and medical support should be need, until an individual fully recovers from a work-related injury [6, 24, 25]. Further studies should prioritize injuries that occur frequently but which are not yet addressed through preventative or management measures.

Among 2154 approved cases, we could analyze more variables for the sex, age, and job duration of 1344 compensated cases (62.4%). However, there are insufficient data on sex, age, and job duration for all compensated cases. As a result, since there is no data on the number of firefighters per job duration, duty, sex, and age, there is a limitation of research that fails to calculate the incidence rate of firefighters by job duration, duty, sex, and age. We could not be concluded that incidence of injury is high or low depending on these characteristics due to missing components of data. 10–20 years’ firefighter, which can be judged to high proficiency, accounts for 48.4% of all in all injuries, suggests that preventive education for the skilled firefighter is needed continuously.

## Conclusion

In summary, as a result of the study, we could obtain the information necessary to establish preventive measures such as cause of accident and region with high accident rates. However, the number of applications for compensated injuries was very small compared to the frequency of injuries found in previous studies. This suggested that many firefighter injuries can become chronic due to lack of appropriate treatment, so it is necessary to introduce an injury monitoring system and improve the accessibility of compensated injuries. In addition, the results of this study can be used to analyze the changing trend of work-related injuries among firefighters and to compare occupational accidents with other occupations in the future; they can also be used as baseline data for establishing work-related injury prevention policies for firefighters.

## Abbreviations

EMS: Emergency medical services
GEPS: Government Employees Pension Service
NFA: National Fire Agency
